# Psychological Coping and Behavioral Adjustment Among Older Adults in Times of COVID-19: Exploring the Protective Role of Working Memory and Habit Propensity

**DOI:** 10.1007/s10804-022-09404-9

**Published:** 2022-05-26

**Authors:** Lotte P. Brinkhof, K. Richard Ridderinkhof, Irene van de Vijver, Jaap M. J. Murre, Harm J. Krugers, Sanne de Wit

**Affiliations:** 1 Department of Psychology, Faculty of Behavioural and Social Sciences, University of Amsterdam, Amsterdam, The Netherlands; 2 Centre for Urban Mental Health, University of Amsterdam, Amsterdam, The Netherlands; 3 Faculty of Science, Swammerdam Institute for Life Sciences, University of Amsterdam, Amsterdam, The Netherlands; 4 Amsterdam Brain & Cognition (ABC), University of Amsterdam, Amsterdam, The Netherlands

**Keywords:** Well-being, COVID-19, Older adults, Habit propensity, Working memory

## Abstract

The impact of the COVID-19 pandemic on mental health, well-being, and behavior is likely influenced by individual characteristics that determine one’s capacity for resilience. In this exploratory study, we examined whether individual differences in working memory (WM) capacity and habit propensity (HP), measured *before* the outbreak, could predict variation in subsequent psychological coping efficacy (as operationalized by measures of depression, mental well-being, perceived stress, and loneliness) and behavioral adjustment (by evaluating compliance and self-reported automaticity of four COVID-19 guidelines) among Dutch older adults (*n* = 36) *during* the pandemic (measured April 25 to May 6, 2020). While we found elevated levels of depression and emotional loneliness, overall mental well-being, and perceived stress were not affected by the pandemic. Contrary to our expectations, we found no robust evidence for a protective role of WM in predicting these outcomes, although our findings hint at a positive relationship with perceived change in mental well-being. Interestingly, WM and HP were found to affect the self-reported automaticity levels of adherence to behavioral COVID-19 guidelines (i.e., washing hands, physical distancing), where a strong HP appeared beneficial when deliberate resources were less available (e.g., low WM capacity). These novel and preliminary findings offer new potential avenues for investigating individual differences in resilience in times of major life events or challenges.

**Supplementary Information:**

The online version contains supplementary material available at 10.1007/s10804-022-09404-9.

## Introduction

Soon after the World Health Organization declared COVID-19 a pandemic, it became clear that older adults were especially susceptible and at higher risk for developing serious complications from infection (e.g., Wang et al., 2020a). Accordingly, governments across the world adopted strict measures to contain the outbreak and protect their elders. This led to severe disruptions of daily routines and lifestyle behaviors (de Haas et al., 2020; Rodríguez-Rey et al., 2020), and for some people to moderate to severe psychological consequences (e.g., increased levels of depression, loneliness, and stress Krendl & Perry, 2020; Rodríguez-Rey et al., 2020; van Tilburg et al., 2020; Wang et al., 2020b). The early impact of the pandemic on mental health, well-being, and behavior in older adults was likely influenced by individual characteristics that determine someone’s capacity for resilience (Polizzi et al., 2020; Uddin, 2021). The present study, conducted during the early stage of the COVID-19 pandemic, was set out to explore the (potentially) protective role of cognitive capacities (in particular, working memory and habit propensity) in psychological coping and behavioral adjustment among older adults.

Resilience is commonly conceptualized as the process of adapting well in the face of difficult or challenging life experiences, especially through mental, emotional, and behavioral flexibility and adjustment to external and internal demands (American Psychological Association, 2020b). It can be seen as a protective characteristic that helps individuals to regain or maintain normal levels of functioning and mental well-being after setbacks (Hayman et al., 2017; Hildon et al., 2010), and can, therefore, strongly impact successful aging (e.g., MacLeod et al., 2016). Indeed, old age is marked by several challenges that require adequate regulation (e.g., physical/cognitive decline), but especially in response to major life events, such as the COVID-19 pandemic, (flexible and) prompt adaptation may be essential to prevent maladaptive developmental changes (Kamo et al., 2011; Shenk et al., 2009). Here, we distinguish between psychological coping and behavioral adjustment as vital elements of resilience. Psychological coping involves the use of cognitive strategies to modulate internal and external demands that are appraised as taxing or exceeding personal resources (Endler & Parker, 1990; Lazarus & Folkman, 1984). Behavioral adjustment refers to the actual changes in behavior that individuals make in accordance with environmental demands (American Psychological Association, 2020a; Kitayama et al., 2018).

**Fig. 1:**
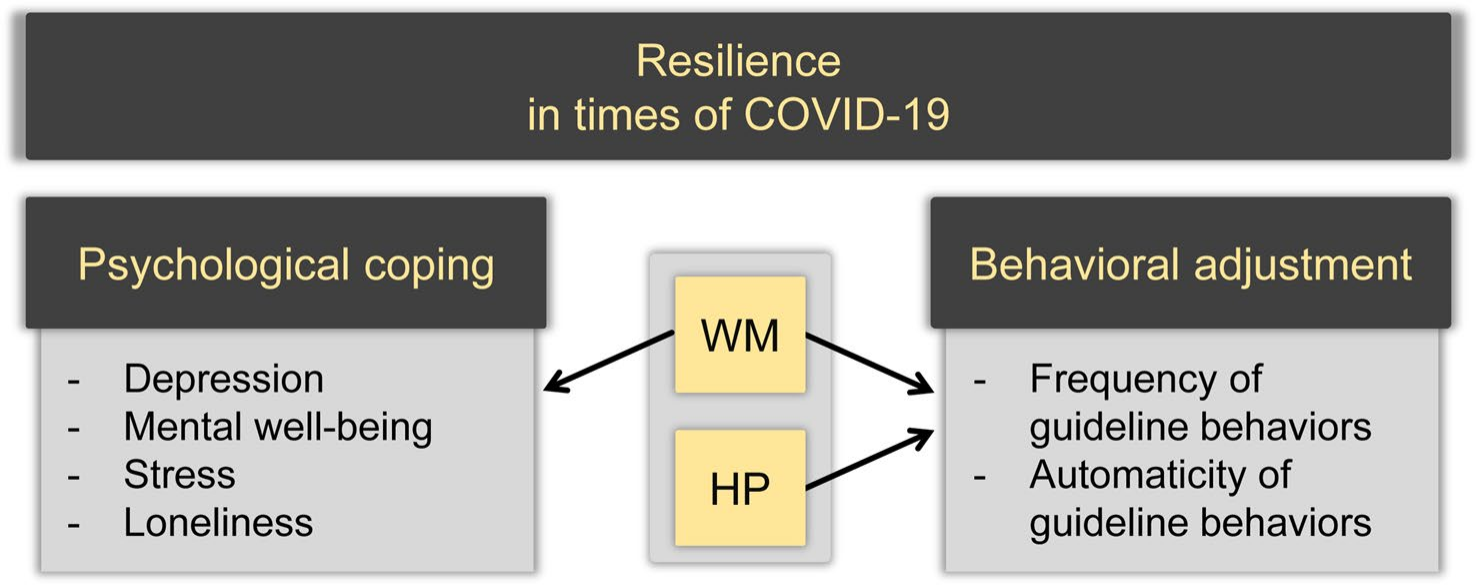
Psychological coping and behavioral adjustment as vital elements of resilience. *Note* This schematic shows how we distinguish psychological coping and behavioral adjustment as vital elements of resilience and illustrates which cognitive factor(s) (both WM and HP, or WM only) we evaluate as potential protective predictor(s) of the outcome variables pertaining to these elements of resilience. *WM* Working memory; *HP* habit propensity

In the present study, we considered the protective role of two aspects of cognitive functioning in resilience: working memory (WM) and habit propensity (HP; only in relation to behavioral adjustment outcomes; see Fig. 1). Working memory (WM) refers to the temporary storage and manipulation of a limited amount of information (Baddeley, 1992; Baddeley & Hitch, 1974) and has been suggested to facilitate resilience by ‘enabling individuals to organize and assimilate information associated with adverse circumstances, to plan and make appropriate decisions to guide behavior, and to regulate emotions, thereby enabling the adaptation to and coping with adversity’ (Bemath et al., 2020, p. 4). Indeed, WM capacity has been shown to be positively correlated with coping (Andreotti et al., 2013; Schmeichel & Demaree, 2010; Schmeichel et al., 2008; Stawski et al., 2010). In turn, adaptive coping strategies have been related to better outcome measures of resilience during COVID-19, including better well-being and reduced mental problems (Dawson & Golijani-Moghaddam, 2020; Passavanti et al., 2021; Pearman et al., 2020). This suggests that people higher in WM capacity may have better resources for coping and maintaining mental health in the face of difficult or challenging life experiences, such as the COVID-19 pandemic. The first aim of our study was to examine whether WM capacity, as assessed prior to the pandemic, could indeed protect individuals from maladaptive changes in mental well-being, depression, loneliness, and stress among older adults during the pandemic (see Fig. 1). In other words, we operationalize the efficacy of coping in terms of measures of well-being and mental health.

Besides its potential role in psychological coping, WM may also facilitate flexible adjustment to *behavioral challenges*, as was required to adequately and promptly incorporate mandated COVID guidelines into one's daily life. WM capacity is essential for the organization of goal-directed behaviors, as it maintains and manipulates task-relevant information and thereby facilitates conscious and deliberate action selection (Hofmann et al., 2008). In line with this idea, previous research during the initial stage of the pandemic showed that individuals high in WM capacity adopted social-distancing rules better as compared to those low in capacity (Xie et al., 2020), and high levels of self-control (i.e., which largely depends on WM) have been associated with better adherence to social-distancing guidelines (Bieleke et al., 2021; Wolff et al., 2020). Furthermore, subjective cognitive functioning was associated with increased adherence to several prevention behaviors (e.g., avoid touching surfaces in public; Thoma et al., 2021).

However, both WM capacity and goal-directed control have been shown to decline with increasing age (Eppinger et al., 2013; Kirova et al., 2015), compromising immediate, flexible behavioral adjustment. This is in line with the age-related deterioration of the prefrontal cortex (PFC; Grieve et al., 2007), with particularly the dorsolateral and ventromedial PFC being implicated in WM and goal-directed control, respectively (Watson et al., 2018). Consequently, in line with dual-process accounts of action control, behavioral change in older adults may be relatively dependent on the gradual automatization of efficient routines (Verplanken, 2006; Wood & Rünger, 2016). Experimental research with the outcome-revaluation paradigm has provided evidence for a disrupted balance between habitual and goal-directed processes in healthy aging. Specifically, older adults showed a higher vulnerability to ‘slips-of-action’ towards no longer valuable outcomes, suggesting a relatively stronger involvement of habitual processes (as opposed to goal directed; de Wit et al., 2014; Watson & de Wit, 2018). This disposition towards habitual (action) control is referred to as *habit propensity* (HP). Indeed, a strong HP may be due to relatively weak goal-directed control in aging, but could also (at least partially) arise as a consequence of stronger habit formation. Here, we argue that a strong habit propensity may facilitate the automatizing of novel guideline behaviors by predisposing individuals to habitual responding (Lally & Gardner, 2013; Linnebank et al., 2018), especially when other cognitive resources that enable flexible adjustment are less available (e.g., low WM capacity, or increased WM load/stress; e.g., Otto et al., 2013; Watson et al., 2021). Therefore, the second aim of our study was to examine whether individual differences in both WM capacity and HP could affect compliance and automatization of COVID-19 guideline behaviors (Fig. 1), as indicators of adequate behavioral adjustment.

**Fig. 2:**
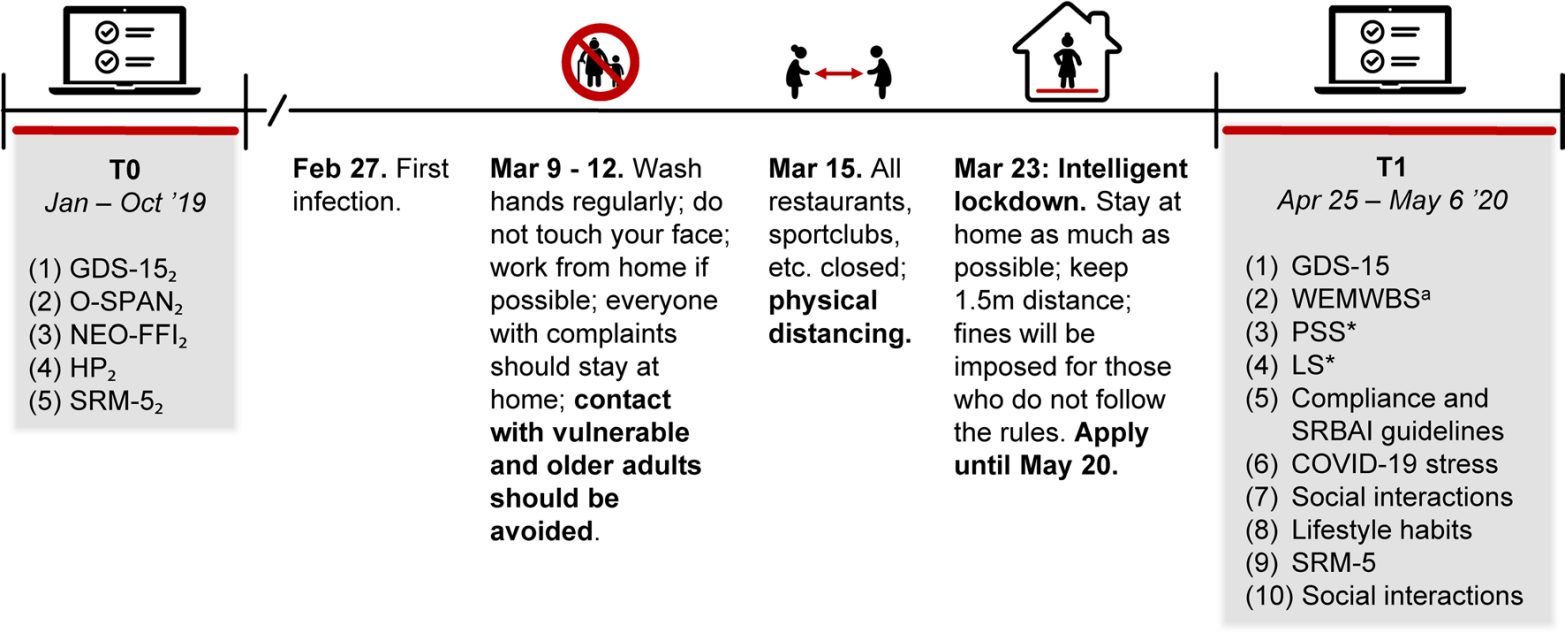
Timing and overview of the current study. *Note* This figure shows the timing of the current study (T1) with respect to the original study (T0) and the COVID-19 related events and guidelines in the Netherlands. Questionnaires and tasks of the original study from which data were included in the current study are also shown, with the numbers in subscript depicting the corresponding lab session. *GDS-15* Geriatric Depression Scale-15; *O-SPAN* Operation Span; *NEO-FFI* NEO Five-Factor Inventory; *HP* Habit Propensity; *SRM-5* Social Rhythm Metric-5; *WEMWBS* Warwick-Edinburgh Mental Well-being Scale; *PSS* Perceived Stress Scale; *LS* Loneliness Scale; *SRBAI* Self-reported Behavioral Automaticity Index. ^a^Participants also reported on the perceived change in the thoughts and feelings as described in the WEMWBS, PSS, and LS

As part of another as of yet unpublished study, we assessed WM and HP in a small (Dutch) sample of older adults prior to the onset of the pandemic. Subsequently, at the start of the pandemic, we recognized a unique opportunity to relate baseline cognitive functioning (that was unaffected by the pandemic) to subsequent resilience, as reflected in both psychological coping and behavioral adjustment. To this end, we obtained self-report measures from (a smaller group) of the same individuals, pertaining to mental health, well-being, and COVID-guideline compliance behavior during the initial lockdown period of the pandemic (Fig. 2). We hypothesized that WM capacity would be a protective factor, as reflected in a positive relationship with change in mental well-being and negative relationship with change in depression, loneliness, and stress. The role of WM and HP in predicting compliance and self-reported automaticity of four main COVID-19 guidelines was assessed in an exploratory fashion.

## Methods

### Participants

A total of 74 community-dwelling older and 71 younger adults participated in a study on prospective memory and routine formation in healthy aging (T0; a future publication of this study will refer to the present paper). Volunteers who gave consent to be approached for future research were subsequently invited to participate in the current study (T1). Forty-one older adults accepted the invitation, of which 36 participants completed the study (7 males, 65–83 years, *M* = 71.1, SD = 4.43). Due to a minimal number of responses from younger individuals, this age group was not considered. All participants were free from severe cognitive decline, as indicated by scores higher than 17 on the Cognitive Screening Test 20 (Deelman et al., 1989; van Toutert et al., 2016), reported no diagnosed neurological or psychiatric disorders, and scored above average on intelligence scores at T0. Participants’ average crystallized intelligence (or IQ) was 118.56, SD = 7.88 (with raw scores: *M* = 92.8, SD = 6.66; see: Dutch Reading Test for Adults; Schmand et al., 1991). The average Wechsler Adult Intelligence Scale (WAIS)-III Matrix Reasoning (MR) score was 15.7, SD = 3.42, and 11.3, SD = 1.85, for the raw and norm scores, respectively (Peck, 1970; Wechsler, 2008). One participant reported to have been tested positive for COVID-19 at T1, but only with minimal symptoms. The study was approved by the local Ethics Review Board of The Faculty of Social and Behavioral Sciences of the University of Amsterdam, and complied with relevant laws and institutional guidelines (2020-CP-12248).

### Procedure

An overview of the timing of the current study with respect to the original study and the COVID-19-related events and guidelines is shown in Fig. 2. The original study consisted of two lab sessions, an intervention phase, and another lab session. For the current study, several measurements that were taken during the original study (in different lab sessions) were used. At T1, participants received an e-mail with a link that provided access to the study and questionnaires were filled out online through Qualtrics (www.qualitrics.com) and the LOTUS platform (www.lab.uva.nl/lotus). Each part took approximately 15 min to complete and included different questionnaires. Participants were forced to answer each question in order to proceed, resulting in no missing questionnaire data at T1. At T0, participants completed all questionnaires and tasks, with the exception of the depression questionnaire (see Materials).

### Materials

#### WM Capacity (T0)

WM capacity was measured with a shorter version of the Operation Span Task (O-SPAN), where participants had to remember sequentially presented words in the correct order while simultaneously solving simple math equations (Turner & Engle, 1989). In the shorter version (based on Oswald et al., 2015), the number of equations and words varied between three and five per set and each set type was presented three times in unpredictable order. The average percentage of correctly remembered words at the correct location were computed per set type, after which the sub-scores were averaged into a total score (Conway et al., 2005).

#### Habit Propensity (T0)

HP was derived from a static version of the Symmetrical Outcome-Revaluation Task (SORT) that participants completed in the initial study (van de Vijver et al., 2021; for a published study with the original version see: Watson et al., 2021). Participants were instructed to earn as many valuable outcomes (certain fruit pictures and addition or subtraction of points from a total score) by pressing a key when their availability was signaled by a discriminative stimulus (different symbols). During the instrumental training, they learned by trial and error which valuable or non-valuable outcomes were signaled by the discriminative stimuli. Subsequently, some of these outcomes were devalued and others upvalued through instruction (i.e., incongruent trials), while others retained their training value (i.e., congruent trials). Participants were subsequently presented with a sequence of discriminative stimuli and had to respond rapidly to stimuli that predicted still-valuable and upvalued outcomes, while refraining from responding to stimuli that signaled still-not-valuable and devalued outcomes. A HP score was operationalized as the mean of the accuracy differences between incongruent trials during which behavior had to be flexibly adjusted and congruent trials (still-valuable—upvalued; still non-valuable—devalued).

#### Depression (T0 and T1)

The Geriatric Depression Scale-15 (GDS-15; *α*: 0.65–0.73) was used to measure (change in) depressive symptomatology (Burke et al., 1991). The GDS-15 consists of 15 questions that can be answered with ‘yes’ or ‘no’ (e.g., ‘Do you feel happy most of the time?’). One point is assigned to each answer that indicates depressive symptomatology (0–15). A total score of 6 hints to the possibility of depression. One of the participants included in the current study did not fill out the GDS-15 at T0 and was, therefore, excluded from all GDS-15 analyses.

#### Mental Well-Being, Loneliness, and Perceived Stress (T1)

The Dutch language version of the 14-item Warwick-Edinburgh Mental Well-being scale (WEMWBS; Tennant et al., 2007; e.g., ‘I have been feeling good about myself’), the 6-item Loneliness Scale (LS; (de Jong-Gierveld & van Tilburg, 2006; e.g., ‘There are plenty of people I can lean on when I have problems’), and the 10-item Perceived Stress Scale (PSS; Cohen et al., 1983; e.g., ‘How often have you been upset because of something that happened'), were used to assess mental well-being, loneliness, and perceived stress, respectively. The WEMWBS and PSS items were scored on Likert scales (1: never, 5: always; 0: never; 4: very often), and summed to a total. Response options for the items of the LS were ‘yes,’ ‘more or less,’ or ‘no,’ with one point being assigned to each answer pointing in the loneliness direction (including ‘more or less’). Total, as well as social and emotional subscale scores were computed. For all constructs, higher scores indicated better mental well-being and more loneliness and perceived stress. The Cronbach’s *α* reliability coefficient of the WEMWBS and PSS were 0.83 and 0.85, respectively. For the LS, the Cronbach’s *α* of the emotional subscale was remarkably low (0.34; as opposed to 0.77 for the social subscale), which negatively impacted the internal consistency of the overall scale (0.45). This could be explained by the fact that agreement with one of the emotional loneliness items (i.e., ‘I miss having people around me') was exceedingly higher than for the other items. Since this pattern of responding was, however, completely in agreement with the anticipated impact of the pandemic (see also: Huber & Seifert, 2022; Landmann & Rohmann, 2021; van Tilburg et al., 2020) and may, therefore, provide highly valuable information, we retained the item and relied on the reliability coefficients from previous studies using the loneliness scale, ranging from 0.80 to 0.90 (de Jong-Gierveld & van Tilburg, 1999). For all constructs, higher scores indicated better mental well-being and more loneliness and perceived stress.

Participants were also asked to rate the thoughts and feelings as described in the WEMWBS (*α*: 0.77), LS (not computed, see previous paragraph), and PSS (*α*: 0.79) over the past two weeks as compared to their normal experience. Each thought or feeling was scored on a 5-point Likert scale, ranging from ‘a lot less than normal’ (1) to ‘a lot more than normal’ (5) with ‘similar to normal’ (3) in the middle, and summed to a total of 14–70, 3–30, and 14–50 points for the different constructs, respectively.

#### Compliance to and Self-reported Automaticity of COVID-19 Guidelines (T1)

Participants were also asked to report to what extent they adhered to four main COVID-19 guidelines: (1) washing hands after coming come, (2) keeping 1.5 m distance in the supermarket, (3) keeping 1.5 m distance outside, and (4) not touching your face; and how many times per week they went outside and to the supermarket. Although both guideline 2 and 3 are related to social distancing, they pertain to two highly distinct contexts, with different levels of challenge (i.e., it is more challenging to keep distance in the supermarket). Hence, we consider them as different types of behaviors. Answers were scored on a 5-point Likert Scale from never (1) to always (5), reflecting the relative frequency of performing the behavior. The number of times/days participants went outside or to the supermarket was also assessed.

The Self-Report Behavioral Automaticity scale (SRBAI; Gardner et al., 2012) was used to assess the automaticity of the behaviors linked to the guidelines (*α*: 0.71–0.87). The behavior of interest was followed by four statements to which participants reported their level of agreement (e.g., ‘Washing my hands after coming home is something I do without thinking’). Each item was scored on a visual analogous scale from 0 to 100 (totally disagree–totally agree), and a mean SRBAI score was calculated for each guideline, with higher scores indicating stronger automaticity.

#### Other Materials

Individual differences in the personality dimension conscientiousness, as assessed with the 12-item NEO Five-Factor Inventory (NEO-FFI) conscientiousness subscale (Costa & McCrae, 1989; e.g., ‘I keep things neat and clean', *α*: 0.82) at T0, and COVID-19 related stress (T1; determined with 10 items selected from a longer list generated by Kalisch et al., 2020; e.g., ‘Having corona symptoms’ or ‘Having less exercise than usual’) were also included. The NEO-FFI was scored on a 5-point Likert Scale (1: strongly disagree–5: strongly agree), and four negatively formulated items were reverse scored prior to summation. For the COVID-related stress list, participants were asked to report how burdensome the stressor was on a scale ranging from ‘not burdensome at all’ (1) to ‘very burdensome’ (5). If they did not experience the stressor, participants could indicate that by selecting option 0 (‘This situation did not occur’). Higher scores indicated more conscientiousness, and COVID-19-related stress. Questions related to lifestyle behaviors and regularity were solely included for exploratory purposes, and results are reported elsewhere [https://doi.org/10.21942/uva.19533502].

### Analyses

#### Design

*Psychological Coping* To determine to what extent depressive symptomatology increased as a result of the COVID-19 pandemic, scores on the GDS-15 at T0 and T1 were compared. Next, a bivariate regression model was adopted to determine whether O-SPAN scores could predict changes (T1–T0) in depressive symptomatology. As difference scores can sometimes be unreliable, we also adopted a multiple regression, with GDS-15 T1 scores being predicted by O-SPAN scores, while correcting for GDS-15 T0 scores. Given the lack of a pre-COVID evaluation, the impact of the pandemic on mental well-being, loneliness, and perceived stress was assessed by comparing sum scores of the WEMWBS, LS, and PSS to their corresponding population norms as assessed prior to the pandemic (51.61, 1.89, 12; Stewart-Brown & Taggart, 2015; de Jong-Gierveld & van Tilburg, 2010; Nordin & Nordin, 2013; also see Fig. 2). Furthermore, self-perceived change in mental well-being, loneliness, and perceived stress were compared to the sum scores reflecting no (average) change in those constructs (conceptual average; 42, 18–9 for subdimensions—and 30, respectively). Three separate bivariate regression models determined to what extent O-SPAN scores were predictive of the self-perceived change in those three constructs.

*Behavioral Adjustment* To ascertain compliance and automaticity of the four guideline behaviors and the extent to which these were predicted by individual differences in O-SPAN and HP scores, several (2 × 4) regression models were adopted with frequency or SRBAI scores included as outcome variable. Models aimed at predicting frequency included SRBAI scores as predictor, and vice versa. Other predictors included in these models were the actual number of times/days someone went outside or to the supermarket, total scores on the PSS and COVID-19-related stressors list and NEO-FFI conscientiousness scores. Conscientiousness was thought to facilitate the formation of new COVID-guideline routines (Conner et al., 2007). Inclusion of the stress variables was based on previous studies showing that stress may influence the balance between goal-directed and habitual control (Schwabe & Wolf, 2010, 2011).

### Statistical Analyses

Statistical analyses were carried out in R 4.0.0 (R Core Team, 2020), with alpha set to 0.005 to identify robust findings and minimize the likelihood of false positives as consequence of multiplicity and still preserve reasonable power (Drachman, 2012).

#### Psychological Coping

Multiple one and two sample *t* tests (paired if possible), or their non-parametric alternatives, were employed to analyze the described comparisons related to psychological coping. Cohen’s *d* and Wilcoxon *r* were used as effect sizes for the parametric and non-parametric *t* test, respectively. For the bivariate regression models, outliers’ points were identified and removed with cooks’ distance, using the traditional 4/n criterion.

#### Behavioral Adjustment

To investigate differences between guidelines in self-reported automaticity, we used mixed-design analyses of variance (ANOVAs), as provided by the R software package ‘afex’ (Singmann et al., 2020), complemented with two-tailed *t* tests. In case an interaction effect was observed, a type III sum of squares (SS) approach was used. Otherwise, we continued with the analysis for main effects, using a type II SS approach (Langsrud, 2003). To make a decision on appropriate *p* value corrections for violations of sphericity we used a Greenhouse–Geisser estimate of sphericity (*ξ*). When *ξ* < 0.75, the Greenhouse–Geisser was selected as the appropriate correction; when *ξ* > 0.75 Huynh–Feldt was used. Partial eta squared (*η*_*p*_^2^) was used as measure of effect size.

Non-parametric relative (guideline) frequency data was tested by means of the non-parametric ANOVA-Type Statistic (ATS; R Software Package: nparLD; Noguchi et al., 2012). This ATS allowed us to measure the relative treatment effects (RTEs) based on the mean ranks (i.e., the probability an observation randomly chosen from the whole dataset is smaller than a randomly chosen observation from that specific grou*p* and/or timepoint), with the denominator degrees of freedom set to infinity. When appropriate, pair-wise comparisons of main effects were performed using the mctp.rm (dependent sample) functions from the ‘nparcomp’ R Software Package (Konietschke et al., 2015), providing robust rank-based, Tukey-type non-parametric contrasts. These contrasts were performed by considering differences between each pair in terms of their RTE, providing a measure of effect size.

In the regression models, many variables could theoretically explain the dependent variable. Because of that, a stepwise model selection based on Akaike Information Criteria (AIC) was performed by using the stepAIC() function (R Software Package ‘MASS'; Venables & Ripley, 2002), to ensure parsimonious models that only included the predictors that were necessary to explain the data. Predictors were iteratively added and removed from the predictive model in order to find the subset of variables resulting in the best performing model (i.e., explaining variance of the dependent variable; lowering prediction error). Prior to the stepwise procedure, we checked for (marginally) significant interactions among predictors and used an hierarchical approach to determine whether the observed interaction(s) improved the fit and explained variance of the model in case this term was included in the final model (i.e., delta R2, AIC, and BIC). Again, outliers were removed with cook’s distance 4/*n* criterion.

## Results

### Psychological Coping

Main descriptives and parameters are shown in Tables 1 and 2. P values lower than 0.05 will be repeated in text.

**Table 1:** Different types of comparisons for depression, and (self-perceived) mental well-being, loneliness, and perceived stress

Outcomes	T0		T1		*t* or *Z*	*p*	Cohen’s *d* or Wilcoxon *r*
	M/Median	SD/Q1:Q3	M/Median	SD/Q1:Q3			
*T0 vs. T1*							
GDS	0	0:1	1	0:2	***Z* = 52.5**	**< 0.005**	***r*** = **− 0.48**
*T1 vs. population norm*							
WEMWBS	–	–	54.9	4.77	***t*(35) = 4.13**	** < 0.001**	***d* = 0.69**
PSS	–	–	10.3	4.78	*t*(35) = − 2.16	0.038	*d* = − 0.36
LS	–	–	2	1:3	*Z* = 352	0.770	*r* = 0.05
Social	–	–	0	0:1	–	–	–
Emotional	–	–	1	1:2	–	–	–
*Perceived change (at T1) vs. conceptual average*							
WEMWBS	–	–	42.2	3.62	*t*(35) = 0.27	0.780	*d* = 2.70
PSS	–	–	30.7	3.62	*t*(35) = 1.37	0.180	*d* = 0.23
LS	–	–	19	18:20	*Z* = 313	0.011	*r* = 0.43
Social	–	–	9	8:9	*Z* = 34	0.140	*r* = − 0.25
Emotional	–	–	10	9:11	***Z* = 287**	** < 0.001**	***r* = 0.66**

This table lists different types of comparisons of our mental health and well-being variables, pertaining to psychological coping, and shows their means and standard deviations or medians and quantiles, in case of non-normally distributed variables (i.e., depression and loneliness scores). Depression (GDS) scores of T0 and T1 were directly compared, whereas mental well-being (WEMWBS), perceived stress (PSS), and loneliness (LS) at T1 were compared to its population norms. Moreover, the self-perceived change in mental well-being, perceived stress, and loneliness were contrasted to their conceptual averages. Significant, robust comparisons (*p* < 0.005) are shown in bold

**Table 2:** Results of the psychological coping (bivariate) regression models with working memory capacity as predictor

Dependent variable (outliers removed)	Predictor	*B*	*B* 95% CI	*SE B*	*β*	*r*	β 95% CI	Fit
Depression (GDS) T1–T0 (2)	WM	0.11	[− 2.34, 2.57]	1.20	0.02	0.02	[− 0.35, 0.38]	R2 = 0.000 [0.00, 0.06] R2 = .184* [0.00, 0.30]
Depression (GDS) T1 (1)	WM	− 0.81	[− 3.12, 1.51]	1.14	− 0.12	− 0.15	[− 0.45, 0.22]	
	GDS T0	0.42*	[0.07, 0.77]	1.14	0.40	0.41	[0.07, 0.74]	
Self-perceived change in loneliness (LS) (1)	WM	2.73	[− 0.18, 5.65]	1.43	0.32	0.32	[− 0.02, 0.65]	R2 = 0.100 [0.00, 0.30]
Self-perceived change in mental well-being (WEMWBS) (2)	**WM**	7.31*	**[1.49, 13.14]**	**2.86**	**0.41**	**0.41**	**[0.08, 0.74]**	**R2 = 0.170 [0.01, 0.38]**
Self-perceived change in perceived stress (PSS) (2)	WM	− 1.79	[− 7.44, 3.85]	2.77	− 0.11	− 0.11	[− 0.47, 0.23]	R2 = 0.013 [0.00, 0.17]

This table shows all test-statistics and relevant parameters of the four psychological coping bivariate regression models, with WM capacity included as predictor. For depression, the output of a secondary model, with GDS scores at T1 as outcome variable, and WM and GDS scores at T0 included as predictors is also shown. The number of outliers is depicted between round brackets (column 1). Significant, yet not robust models (*p < 0.05) are shown in bold. B represents unstandardized regression weights, β indicates the standardized regression weights. In case of bivariate regressions, the Pearson’s correlation coefficient (r) is similar to β. Square brackets are used to enclose the lower and upper limits of a confidence interval (CI)

Most participants (*N* = 34) reported relatively low scores on the GDS-15 at T0 and T1. Only two participants had a score above 5 on one of the time points (scores of 6 at T0 and 11 at T1, respectively), indicative of depression. Importantly, the median GDS-15 score at T1 was significantly higher than the median GDS-15 score at T0, *p* = 0.0045, suggesting that depressive symptomatology among older adults increased during the initial phase of the pandemic. O-SPAN scores did not affect the change in depressive symptomatology.

In contrast, WEMWBS scores at T1 were significantly higher than population norms, *p* < 0.001, which was not the case for PSS and LS. However, we found no robust evidence for an overall effect of the pandemic on self-perceived change in mental well-being, perceived stress, and feelings of loneliness, *p* = 0.011, as the mean/median scores of self-perceived change in the thoughts and feeling as described in the WEMWBS, PSS, and LS were not convincingly higher than the conceptual averages (see Supplement A for separate evaluations of WEMWBS and PSS items). The median score of self-perceived change in *emotional* loneliness specifically was, however, significantly higher than 9, *p* < 0.001, indicating that this might indeed have changed. Finally, self-perceived change in WEMWBS, PSS, and LS was not significantly predicted by O-SPAN scores. A positive relationship between O-SPAN scores and self-perceived change in mental well-being was found, *p* = 0.015, albeit not robustly.

To summarize, older adults reported an increase in depressive symptomatology and self-perceived emotional loneliness during the pandemic. WM capacity was not robustly predictive of the pandemic’s effect on depression, mental well-being, loneliness, and perceived stress.

### Behavioral Adjustment

In total, 33 participants completed all measures (except frequency and automaticity for physical distancing in the supermarket, see legend of Table 3) that were, therefore, included in the regression models to predict interindividual variation in compliance and self-reported automaticity. The median/average scores on these predictors are also shown in Table 3. Interestingly, we found a main effect of type of guideline for frequency, ATS: *F*(2.06, *∞*) = 35.429, *p* < 0.001, RTE = 0.50, but this effect was not robust for automaticity scores, *F*(2.03,61.00) = 5.09, *p* = 0.009, *η*_p_^2^ = 0.15. Participants reported relatively low compliance with the guideline to refrain from touching one’s face as compared to other guidelines, all *ps* < 0.001, RTEs < − 0.84. Pre-planned contrasts also revealed relatively lower automaticity levels for this guideline, although not meeting our stringent criteria, all *ps* < 0.028, *d’s* > 0.61. Compliance of hand washing was relatively high as compared to the other guidelines, albeit not robustly, all *ps* < 0.045, RTEs < − 0.29. The frequency of keeping distance from others outside versus in the supermarket appeared to be relatively similar, *p* = 0.74, RTE = − 0.07.

**Table 3:** The median or averages scores of the predictors of the behavioral adjustment regression models

Predictors	M/Median	SD/Q1:Q3
Self-reported automaticity		
Hand washing	66.8	26.7
Physical distance supermarket^a^	70.8	23.3
Physical distance outside	68.9	24.0
Not touching face	53.5	20.7
Relative frequency		
Hand washing	5	5:5
Physical distance supermarket^a^	5	4:5
Physical distance outside	5	4:5
Not touching face	3	3:4
Times outside	8.48	5.47
Days in the supermarket	3.36	2.00
WM	0.52	0.18
HP	32.9	20.2
PSS	9.88	4.76
Conscientiousness	45.6	5.73
COVID-19 stress	18.1	5.06

This table shows the average/median scores of the predictors that are included in the behavioral adjustment regression models, directed at four main COVID-19 guidelines. Medians are shown in case of non-normally distributed variables (i.e., relative frequency). N = 33. WM: Working Memory, HP: Habit Propensity, PSS: Perceived Stress, Relative frequency: scores on a 5-point Likert Scale from never (1) to always (5), reflecting the relative frequency of performing the behavior described in the guidelines

^a^Scores for maintaining physical distance from others in the supermarket are based on 31 participants, since 2/33 did not go to the supermarket at all

Since we found little variation in compliance (per guideline) among participants, we could not properly adopt regression models to predict the frequency. Hence, we only performed the intended analyses on self-reported automaticity. The test statistics and parameters of the final models based on the stepwise model selection procedures are shown in Table 4. Considering our exploratory approach, only *p* values lower than 0.005 will be repeated in text.

**Table 4:** Results of the behavioral adjustment regression models

Predictor	*B*	*B* 95% CI	*SE B*	*β*	*β* 95% CI	*r*	Fit
Washing hands (1)							R2 = 0.700*** [0.33, 0.76] delta R2 = 0.233*** [0.04, 0.43] delta AIC = − 16.41 delta BIC = − 14.94
Relative frequency	27.98***	**[11.61, 44.36]**	**7.93**	**0.46**	**[0.19, 0.73]**	0.58***	
Times outside	− 1.27	[− 2.58, 0.05]	0.64	− 0.24	[− 0.48, 0.01]	− 0.03	
HP	2.87***	**[1.52, 4.21]**	**0.65**	**2.15**	**[1.14, 3.16]**	**0.28**	
WM	132.55**	[42.46, 222.64]	43.6	0.88	[0.28, 1.48]	− 0.24	
HP*WM	− 4.64***	**[0.05, 3.29]**	**0.79**	**− 1.91**	**[− 2.83, − 1.00]**		
PSS	1.67*	[0.25, 2.64]	0.68	0.28	[0.01, 0.55]	− 0.19	
Conscientiousness	1.45*	[− 6.86, − 2.42]	1.07	0.30	[0.05, 0.55]	0.18	
Distance supermarket^a^ (1)							R2 = 0.502*** [0.19, 0.65]
Relative frequency	22.22***	**[9.29, 35.15]**	**6.30**	**0.49**	**[0.20, 0.78]**	**0.36**	
HP	0.73***	**[0.40, 1.07]**	**0.16**	**0.63**	**[0.34, 0.91]**	0.52***	
Distance outside (2)							R2 = 0.496** [0.13, 0.62] delta R2 = .161** [− 0.03, 0.36] delta AIC = − 6.57 delta BIC = − 5.13
Relative frequency	52.23***	**[23.41, 81.05]**	**14.02**	**1.08**	**[0.49, 1.68]**	0.42*	
Times outside	17.64*	[3.73, 31.55]	6.77	3.50	[0.74, 6.26]	− 0.30	
Relative frequency*times outside	4.24**	[− 7.26, − 1.21]	1.47	− 3.74	[− 6.41, − 1.07]		
COVID-19 related stress	1.74*	[− 3.12, − 0.35]	0.67	− 0.39	[− 0.69, − 0.08]	− 0.23	
Not touching face (0)							R2 = 1.03 [0.00, 0.31]
Conscientiousness	− 1.16	[− 2.41, 0.10]	0.61	− 0.32	[− 0.67, 0.03]	0.32	

This table lists the test statistics and relevant parameters of the final behavioral adjustment regression models, after stepwise selection, predicting self-reported automaticity of four main COVID-19 guidelines. The number of outliers are depicted between brackets, and significant, robust predictors (***p < 0.005) are shown in bold. *p < 0.05, **p < 0.01. B represents unstandardized regression weights, β indicates the standardized regression weights. In case of bivariate regressions, the Pearson’s correlation coefficient (r) is similar to β. Square brackets are used to enclose the lower and upper limits of a confidence interval (CI). In case significant interactions were detected, only the output of the second block (with interaction term) is reported, with delta R2 showing the additional explained variance of that model, and delta AIC and delta BIC showing a better fit for the interaction models. *WM* Working Memory, *HP* Habit Propensity, *PSS* Perceived Stress, Relative Frequency: scores on a 5-point Likert Scale from never (1) to always (5), reflecting the relative frequency of performing the behavior described in the guidelines

^a^The model for maintaining physical distance from others in the supermarket only includes the 31 out of 33 participants that went to the supermarket

We found evidence for a positive and robust association between the mean automaticity of keeping physical distance from other in the supermarket and the frequency of performing this action, *p* = 0.002. Moreover, a positive association for this guideline was found with HP, *p* < 0.001, indicating higher automaticity of distancing in participants with a relatively strong habit propensity.

**Fig. 3:**
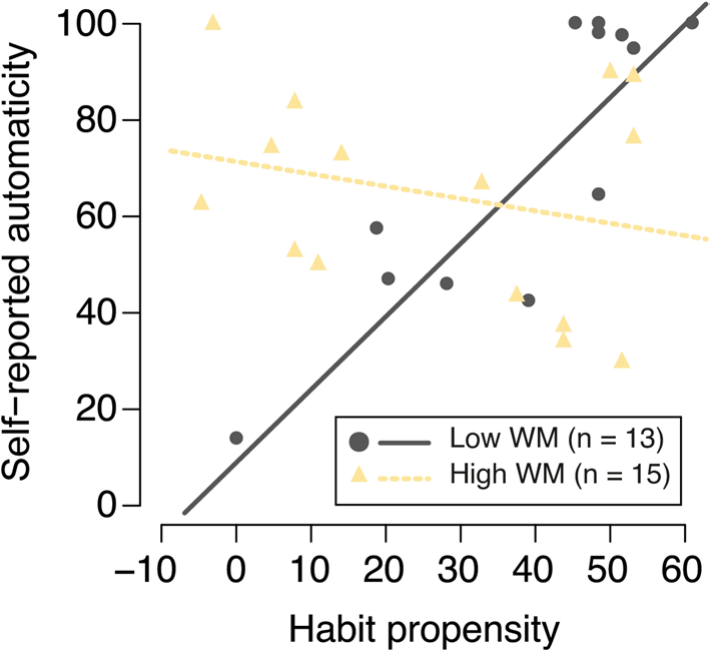
Relationship between self-reported automaticity for hand washing and habit propensity among individuals with different WM capacity. *Note* This figure illustrates the relationship between self-reported behavioral automaticity scores for hand washing and habit propensity among individuals with low (circle, solid line) and high (triangle, dashed line) WM capacity

Similarly, the mean automaticity score for hand washing was significantly predicted by the frequency,* p* = 0.002. Most interestingly, however, the HP*WM interaction term was found to be a significant predictor as well, *p* < 0.001. Two separate linear regression models for participants with low (< 0.45, *n* = 13) and high (> 0.50, *n* = 15, excluding four participants with an O-SPAN score in between these boundaries to ensure a reasonable distinction) O-SPAN scores revealed that the mean automaticity score was positively predicted by the general tendency to rely on habits among participants with relatively low WM capacity, *B* = 1.46, SE = 0.26, 95% CI [0.88, 2.05], *β/r* = 0.87, *p* < 0.001, *R*^2^ = 0.756, 95% CI [0.33, 0.86], but not among participants with relatively high WM capacity, *B* = − 0.26, SE = 0.27, 95% CI [− 0.84, 0.33], *β/r* = − 0.25, *p* = 0.360, *R*^2^ = 0.065, 95% CI [0.00, 0.36] (see Fig. 3). Importantly, the mean automaticity scores of both WM groups were similar, *t*(25) = 0.74, *p* = 0.46 (low WM: *M* = 71.8, SD = 30.1, high WM: *M* = 64.4, SD = 22.1).

Finally, the frequency appeared to be the only factor that could robustly predict the mean automaticity score for keeping physical distance from others outside, *p* < 0.001, and we found no variables that could predict automaticity for refraining from touching one’s face.

In summary, we found that behavioral repetition (as reflected in relative frequency) was predictive of self-reported automaticity of hand washing and physical distancing. Furthermore, the self-reported automaticity of maintaining physical distance in the supermarket was predicted by HP irrespective of WM capacity. This was also the case for hand washing, but only among those relatively low in WM capacity.

## Discussion

The present study examined the impact of individual differences in WM capacity and HP on resilience among older adults during the initial phase of the COVID-19 pandemic. We distinguished between psychological coping and behavioral adjustment as vital elements of resilience. The idea that WM capacity plays an important role in psychological coping and, therefore, prevents maladaptive changes did not receive strong support. While the pandemic did lead to an increase in depressive symptomatology and loneliness, we found that individual differences in WM did not robustly predict changes in depression, loneliness, nor stress in our sample. There was a trend for a positive relation with mental well-being, but this failed to meet our stringent criteria. On the other hand, this study provides unique exploratory insights into the role of WM and HP in behavioral adjustment, as HP was positively related with self-reported automaticity of physical distancing in the supermarket, as well as automaticity of hand washing among those low in WM capacity.

Foremost, this study confirms that the COVID-19 pandemic had an impact on mental health, as reflected in an increase in depressive symptomatology and self-perceived emotional loneliness, in accordance with several other studies conducted during the initial phase of the pandemic (e.g., Luchetti et al., 2020; van Tilburg et al., 2020). Indeed, the design of our study does not allow us to rule out with certainty that increases in these outcomes were in fact due to natural fluctuations. Yet, it seems unlikely that all participants would show a similar pattern of fluctuations that would lead to such main effects. Importantly, since our T0 measurements were taken at completely different times throughout the pre-COVID year, any seasonal effects are highly improbable. Therefore, we argue that the pandemic is indeed the most plausible explanation for the observed increases in depression and loneliness. In contrast, we found that self-perceived mental well-being remained roughly the same (see also: van Tilburg et al., 2020), as did perceived stress. These findings may be explained by the fact that some thoughts and feelings as described in these constructs seemed to be positively affected (e.g., more interested in others), whereas others were negatively affected by the pandemic (e.g., less experienced feelings of optimism; less grip on things; see Supplement A), thereby balancing each other out. It remains to be established whether this pattern would remain stable for a longer period of time.

Although we did not find robust evidence for a role of WM in predicting resilience to the impact of the pandemic on mental health and well-being, one marginal finding is worth mentioning. Specifically, we found a trend for a positive relationship between WM capacity and change in self-perceived mental well-being. While we decreased the evidentiary standard to minimize the likelihood of false positives, this has increased the chance of missing a genuine effect (Drachman, 2012), especially given the limited number of participants we could include in our study (Curran-Everett, 2017). Additionally, it may be that our measure of WM does not tap onto the crucial components that impact psychological coping (see e.g., Fellman et al., 2020). Both leave open the possibility that WM capacity may indeed protect against the pandemic’s effects on psychological health (see e.g., Brush, 2021; Sin et al., 2021). This could be mediated by the role of WM capacity in coping (e.g., Stawski et al., 2010), with (mal)adaptive coping strategies predicting mental health and well-being outcomes during COVID-19 (e.g., Pearman et al., 2020). Alternatively, WM may promote resilience via other higher-order abilities (e.g., attentional processing) that could influence the capacity to withstand difficult life events (Derakhshan, 2020; Johnson et al., 2013). Clearly, these speculations warrant future investigations, in which the relationships between those factors could be estimated using more causal or associative analysis techniques in a larger sample (e.g., network analysis; Brinkhof et al., 2021).

The most compelling factor in explaining variation in behavioral adjustment appeared to be HP. While an overreliance on habits is often linked to clinical conditions (Robbins et al., 2012; Watson & de Wit, 2018), our data suggests that, at a non-clinical level, a disposition towards habit learning may be beneficial. HP was positively related to self-reported automaticity for the maintenance of physical distance in the supermarket and hand washing, albeit the latter only among individuals with relatively low WM capacity. This indicates that a stronger reliance on habitual control can facilitate the gradual automatization of new routines (Lally & Gardner, 2013). In addition, it also indicates that such a disposition may be especially beneficial when more deliberate resources to direct action in accordance with new goals (i.e., reducing transmission, not becoming infected; e.g., Xie et al., 2020) are less available, and the dependence on habitual behavior is enhanced. This applies to those having a relatively low WM capacity in general, as well as to a specific situation in which someone’s WM capacity is taxed with other tasks or strains (e.g., searching for products in the supermarket; maneuvering between other people; Foerde et al., 2006; Otto et al., 2013; Quaedflieg et al., 2019; Watson et al., 2021). In other words, a strong HP may offer an alternative route to automatization and compliance to new behaviors when goal-directed resources cannot be deployed optimally. This is in line with the notion that relying on beneficial habits may be a promising pathway to reduce the need for effortful top-down control functions (Galla & Duckworth, 2015). Moreover, it concurs with recent findings showing that engagement in behavior regulation strategies that are thought to promote the formation of desirable habits (i.e., if–then planning) is associated with more successful and effortless social-distancing adherence, particularly in the long run (Bieleke et al., 2021). Critically, this previous study showed that a strong inclination to engage in such planning facilitated social distancing, especially for those who perceived adherence as difficult. In our study, self-reported automaticity of maintaining physical distance outside may not have been affected by HP (nor WM), because it is relatively easy to comply to this guideline. Altogether, our results emphasize that in response to changes in circumstances and *challenging* environmental demands, older adults may benefit from a stronger propensity to rely on habits or from interventions that facilitate habit formation. Recent insights suggest this also has positive effects for the long term, with Gillebaart et al. (2022) showing that a strong hand washing habit buffered against a decrease in hand washing compliance over time.

We also show that compliance to (and possibly also the automatization of) the guideline to refrain from touching one’s face was lower as compared to the other guidelines. This is in line with the idea that forming new desirable habits is substantially different from breaking unwanted habits and that attaining control over habitual responses can be extremely difficult (Bargh, 1994). Furthermore, the average self-reported automaticity score of refraining could not be predicted by the relative frequency, HP, WM capacity or any other variable. Potentially, when a new desired behavior has to compete with an old habit, a strong HP may actually enhance the expression of the old habit and thereby impedes the formation of a new routine (Quinn et al., 2010). In other words, the contribution of HP to the automatization of new behaviors may depend on the presence and strength of an existing, conflicting habit. Future studies are needed to identify the specific situations and circumstances in which HP may or may not be beneficial when forming new routines.

In line with the idea that being more organized, careful, dependable, self-disciplined, and achievement oriented can reduce the intention–behavior gap (Conner et al., 2007), conscientiousness seemed to have a positive effect on guideline adherence, yet only for hand washing and not robustly so. Similarly, no robust influences of perceived stress or COVID-19 related stress were observed. Interestingly, however, perceived stress seemed to have a positive effect on adherence (hand washing), whereas COVID-19 related stress had a negative effect (distance outside). This suggests that moderate levels of stress may be beneficial, for instance by making people more aware of the importance of hand washing. The negative effect of COVID-19 related stress is in line with the idea that stress can improve reliance on existing habits, thereby impeding the automatization of new, conflicting behaviors (e.g., Schwabe & Wolf, 2010). However, it should be noted that this relationship may also be found because those with more COVID-19 related stress considered their own adherence to be less sufficient and adequate as compared to individuals that were not experiencing too much stress.

The present study pioneers research into the combined role of WM and HP in behavioral adjustment and influence of WM on psychological coping among older adults during a subsequent shared major life event. Despite the small-scale, exploratory character of this study, our findings and individually focused research approach provide a unique window for future research on resilience and the role of individual differences in response to major (historical) life events specifically. A recommendation for follow-up studies is to include more heterogeneous samples that allow for the generalization of current findings. The sample in the present study was above average in intelligence, which may have impacted the results (in particular regarding our cognitive factors, e.g., Conway et al., 2002). Furthermore, most participants were from (municipalities near) Amsterdam, and there may be substantial differences in psychological coping and behavioral adjustment during the pandemic with individuals living in rural areas (e.g., due to higher presence of green space; physical distancing being more challenging in densely populated areas). Another opportune direction for future research would be to explore the role of several *risk factors* in both elements of resilience.

Finally, individuals having undergone a moderate amount of life adversity have been shown to be less vulnerable to the deleterious effects of future stressors than individuals with limited or massive life-time exposure, a phenomenon called ‘stress inoculation’ (Seery et al., 2013; Serino et al., 2014). Similarly, life events, such as the COVID-19 pandemic, may expand individuals’ repertoire of skills and competencies that enable better (flexible) behavioral adjustment in future circumstances. In this way, the COVID-19 pandemic may also bolster against age-related cognitive impairments, thereby reducing overall cognitive aging (Kalisch et al., 2017). Future interventional studies could test whether emotional resilience inoculation, as well as acquired behavioral adjustment skills, may indeed decelerate cognitive aging, and to what extent HP and WM can modulate these effects.

Altogether, this study offered a unique opportunity to examine (potential) protective factors that could determine one’s ability to successfully adapt to challenging and/or difficult life experiences. Specifically, our design enabled us to include baseline levels of WM and HP, thereby excluding the possibility that these cognitive capacities would be affected by the consequences of the pandemic itself (e.g., stress; Klein & Boals, 2001). In this way, we have provided preliminary evidence that WM may be an important resilience factor, explaining variation in behavioral adjustment and possibly also in psychological coping. Especially when more deliberate resources are less available and no conflicting habit is present, a high propensity to rely on habitual control also seems to contribute to behavioral adaptability. While future work is needed to test the robustness of our findings, the present study offers novel insights into the cognitive components that may facilitate resilience and offers new potential avenues to investigate individual differences in resilience in times of shared major life events (such as the COVID-19 pandemic), as well as other (age-related) challenges (e.g., loss of a spouse, physical decline). Moreover, our results highlight the potential of lifestyle interventions and strategic planning techniques (i.e., implementation intentions) that capitalize on habits to improve prompt and adequate behavioral adjustment in favor of resilience among older adults.

## Supplementary Information

Below is the link to the electronic supplementary material.

Supplementary file1 (PDF 40 KB)

## Data Availability

The data (and codes) that support the findings of this study are available upon reasonable request, via https://doi.org/10.21942/uva.14519475.

## Declarations

### Copyright Statement

The Warwick–Edinburgh Mental Well-being Scale was funded by the Scottish Government National Programme for Improving Mental Health and Well-being, commissioned by NHS Health Scotland, developed by the University of Warwick and the University of Edinburgh, and is jointly owned by NHS Health Scotland, the University of Warwick and the University of Edinburgh.
