# Supplementary material for: Psychological Coping and Behavioral Adjustment Among Older Adults in Times of COVID-19: Exploring the Protective Role of Working Memory and Habit Propensity
Source: J Adult Dev. 2022 May 26;29(3):240–54. doi: 10.1007/s10804-022-09404-9 (PMC9132676; doi:10.1007/s10804-022-09404-9)
Supplement: Supplementary file 1 — Supplementary file1 (PDF 40 KB) [file 10804_2022_9404_MOESM1_ESM.pdf]

[Supplementary Methods & Results, A] Psychological coping and behavioral adjustment among Older Adults in Times of COVID-19: Exploring the Protective Role of Working Memory and Habit Propensity

Supplemental results

Psychological coping

In addition to the main analyses regarding loneliness, we also performed some exploratory analyses to determine the effect of living with or without a partner. Interestingly, living alone or with someone else did not significantly affect total loneliness, U = 155.5 p = .53, r = .11, nor the two subdimensions separately, social: U = 128.5, p = .73, r = -.06; emotional: U = 158.5, p = .45, r = .13. However, older adults living alone did report a change in perceived loneliness, as indicated by an average self-perceived change score of 18.8 (SD = 1.58) being higher than 18, t(24) = 2.53, p = .018, d = 0.51, although not robustly. For older adults living together (median = 19, *Q1:Q3* = 18:19), no significant effect was found, U = 27, p = .22, r = .39. The (minimal) effect among older adults living alone seemed mainly driven by a self-perceived change in emotional loneliness. The scores of self-perceived change in social loneliness were not significantly different from 9, Z = 9.5, p = .25, r = -.24 (median = 9, *Q1:Q3* = 9:9), while the scores of self-perceived change in emotional loneliness were significantly higher (M = 10.2, SD = 1.28), t(24) = 4.53, p < .001, d = 0.91. Although we did not find a significant result for older adults living together when looking at the self-perceived change in total loneliness, our result hint to an increase in emotional loneliness in this subgroup, t(10) = 3.07, p = .012, d = .92, but again not robustly. Feelings of social loneliness were also not significantly changed during the corona pandemic in this subgroup, U = 7, p = .25, r = -.37 (median = 8, *Q1:Q3* = 8:9).

Self-perceived change in mental well-being was not significantly different from 42, as reported in the main text. However, the scores on the individual items were substantially different from each other, $F(6.36, 222.6) = 5.71$, $p < .001$, $\eta p^2 = 0.14$. For that reason, we also performed separate one-sample Wilcoxon-Signed Rank tests for the scores on the individual items. Here, a score of 3 indicated no overall change in that particular thought or feeling. This exploratory analysis revealed that participants reported less experienced feelings of optimism (median = 3, *Q1:Q3* = 2:3), $Z = 15$, $p = .008$, $r = -0.45$, and usefulness (median = 3, *Q1:Q3* = 2:3), $Z = 13$, $p = .014$, $r = -0.42$, but they were more interested in others (median = 3, *Q1:Q3* = 3:4), $Z = 84.5$, $p = .0031$, $r = 0.5$, and they felt a stronger bond (median = 3, *Q1:Q3* = 3:4), $Z = 91$, $p = .0085$, $r = 0.45$. Indeed, these p-values do not provide robust evidence.

Similar to the WEMWBS, the scores on the individual items of the PSS were substantially different, $F(6.37, 222.91) = 5.48$, $p < .001$, $\eta p^2 = 0.14$. Hence, we performed separate one-sample t-tests for the scores on the individual items for this scale as well. Here, a score of 3 indicated no overall change in that particular item or component of perceived stress. This exploratory analysis revealed that participants reported to feel like things were going significantly less like they wanted them to be (median = 3, *Q1:Q3* = 3:4), $Z = 135$, $p = .002$, $r = 0.53$, and to feel like they had significantly less grip on things (median = 3, *Q1:Q3* = 3:4), $Z = 104$, $p < .005$, $r = 0.47$. The other items/components of perceived stress were not significantly different from 3.
